# Supplementary material for: Association of Serum 25(OH)D with Metabolic Syndrome in Chinese Women of Childbearing Age
Source: Nutrients. 2022 May 30;14(11):2301. doi: 10.3390/nu14112301 (PMC9182986; doi:10.3390/nu14112301)
Supplement: Supplementary file 1 [file nutrients-14-02301-s001.zip › nutrients-1720289-supplementary.pdf]

Table S1. Hardy Weinberg testing results of vitamin D metabolism related gene polymorphisms.

Parameters	rs12794714					rs2282679					rs2228570				
	AA	GA	GG	MAF	*P*	GG	GT	TT	MAF	*P*	AA	GA	GG	MAF	*P*
District															
Eastern	73(14.0)	256(49.0)	193(37.0)	0.39	0.458	49(9.4)	223(42.7)	250(47.9)	0.31	0.978	109(20.9)	250(47.9)	163(31.2)	0.45	0.509
Central	73(15.2)	232(48.4)	174(36.3)	0.39	0.818	59(12.3)	205(42.8)	215(44.9)	0.34	0.395	104(21.7)	249(52.0)	126(26.3)	0.48	0.397
Western	73(14.4)	223(44.1)	210(41.5)	0.36	0.305	44(8.7)	193(38.1)	269(53.2)	0.28	0.309	121(23.9)	249(49.2)	136(26.9)	0.49	0.787
Region															
Northern	107(15.1)	346(48.7)	257(36.2)	0.39	0.632	74(10.4)	307(43.2)	329(46.3)	0.32	0.901	143(20.1)	341(48.0)	226(31.8)	0.44	0.523
Southern	112(14.1)	365(45.8)	320(40.2)	0.37	0.670	78(9.8)	314(39.4)	405(50.8)	0.30	0.157	191(24.0)	407(51.1)	199(25.0)	0.50	0.581
Area type															
Urban	97(15.8)	279(45.4)	238(38.8)	0.39	0.346	64(10.4)	263(42.8)	287(46.8)	0.32	0.799	135(22.0)	301(49.0)	178(29.0)	0.47	0.761
Rural	122(13.7)	432(48.4)	339(38.0)	0.38	0.430	88(9.9)	358(40.1)	447(50.0)	0.30	0.214	199(22.3)	447(50.1)	247(27.7)	0.47	0.943
Season															
Spring	25(21.2)	55(46.6)	38(32.2)	0.45	0.636	11(9.3)	51(43.2)	56(47.5)	0.31	0.945	23(19.5)	60(50.8)	35(29.7)	0.45	0.868
Autumn	108(14.0)	368(47.5)	298(38.5)	0.38	0.785	81(10.5)	322(41.6)	371(47.9)	0.31	0.408	179(23.1)	382(49.4)	213(27.5)	0.48	0.800
Winter	86(14.0)	288(46.8)	241(39.2)	0.37	0.947	60(9.8)	248(40.3)	307(49.9)	0.30	0.381	132(21.5)	306(49.8)	177(28.8)	0.46	0.958
Nationality															
Han	185(14.1)	632(48.2)	493(37.6)	0.38	0.467	137(10.5)	553(42.2)	620(47.3)	0.32	0.436	293(22.4)	658(50.2)	359(27.4)	0.48	0.828
Ethnic minorities	34(17.3)	79(40.1)	84(42.6)	0.37	0.365	15(7.6)	68(34.5)	114(57.9)	0.25	0.358	41(20.8)	90(45.7)	66(33.5)	0.44	0.372
Age (years)															
18-25	76(15.4)	220(44.6)	197(40.0)	0.38	0.297	51(10.3)	205(41.6)	237(48.1)	0.31	0.554	123(24.9)	233(47.3)	137(27.8)	0.49	0.258
25-35	81(15.6)	249(47.9)	190(36.5)	0.40	0.969	53(10.2)	214(41.2)	253(48.7)	0.31	0.487	112(21.5)	277(53.3)	131(25.2)	0.48	0.145
35-45	62(12.6)	242(49.0)	190(38.5)	0.37	0.297	48(9.7)	202(40.9)	244(49.4)	0.30	0.569	99(20.0)	238(48.2)	157(31.8)	0.44	0.658
BMI															
Underweight	18(20.6)	66(48.5)	42(30.9)	0.45	0.402	18(13.2)	57(41.9)	61(44.9)	0.34	0.513	34(25.0)	65(47.8)	37(27.2)	0.49	0.702
Normal weight	118(14.0)	395(47.0)	328(39.0)	0.38	0.985	79(9.4)	338(40.2)	424(50.4)	0.30	0.364	188(22.4)	404(48.0)	249(29.6)	0.46	0.348
Overweight	54(14.3)	178(47.2)	145(38.5)	0.38	0.967	37(9.8)	158(41.9)	182(48.3)	0.31	0.824	77(20.4)	204(54.1)	96(25.5)	0.47	0.116
Obesity	19(12.4)	72(47.1)	62(40.5)	0.36	0.887	18(11.8)	68(44.4)	67(43.8)	0.34	0.960	35(22.9)	75(49.0)	43(28.1)	0.47	0.923
Total	219(14.5)	711(47.2)	577(38.3)	0.38	0.966	152(10.1)	621(41.2)	734(48.7)	0.31	0.242	334(22.2)	748(49.6)	425(28.2)	0.47	0.917
